# Effects of Salinity Stress on Chloroplast Structure and Function

**DOI:** 10.3390/cells10082023

**Published:** 2021-08-07

**Authors:** Abdul Hameed, Muhammad Zaheer Ahmed, Tabassum Hussain, Irfan Aziz, Niaz Ahmad, Bilquees Gul, Brent L. Nielsen

**Affiliations:** 1 Dr. M. Ajmal Khan Institute for Sustainable Halophyte Utilization, University of Karachi, Sindh 75270, Pakistan; ahameed@uok.edu.pk (A.H.); mzahmed@uok.edu.pk (M.Z.A.); thussain@uok.edu.pk (T.H.); irfanaziz@uok.edu.pk (I.A.); bilqueesgul@uok.edu.pk (B.G.); 2 Agricultural Biotechnology Division, National Institute for Biotechnology & Genetic Engineering (NIBGE), Faisalabad 44000, Pakistan; niazbloch@yahoo.com; 3 Department of Biotechnology, Pakistan Institute of Engineering and Applied Science (PIEAS), Islamabad 44000, Pakistan; 4 Department of Microbiology & Molecular Biology, Brigham Young University, Provo, UT 84602, USA

**Keywords:** salinity stress, photosynthesis, chloroplast, plastid, osmolytes, osmotic adjustment

## Abstract

Salinity is a growing problem affecting soils and agriculture in many parts of the world. The presence of salt in plant cells disrupts many basic metabolic processes, contributing to severe negative effects on plant development and growth. This review focuses on the effects of salinity on chloroplasts, including the structures and function of these organelles. Chloroplasts house various important biochemical reactions, including photosynthesis, most of which are considered essential for plant survival. Salinity can affect these reactions in a number of ways, for example, by changing the chloroplast size, number, lamellar organization, lipid and starch accumulation, and interfering with cross-membrane transportation. Research has shown that maintenance of the normal chloroplast physiology is necessary for the survival of the entire plant. Many plant species have evolved different mechanisms to withstand the harmful effects of salt-induced toxicity on their chloroplasts and its machinery. The differences depend on the plant species and growth stage and can be quite different between salt-sensitive (glycophyte) and salt-tolerant (halophyte) plants. Salt stress tolerance is a complex trait, and many aspects of salt tolerance in plants are not entirely clear yet. In this review, we discuss the different mechanisms of salt stress tolerance in plants with a special focus on chloroplast structure and its functions, including the underlying differences between glycophytes and halophytes.

## 1. Introduction

Soil quality in many parts of the U.S. and worldwide is susceptible to a variety of stresses, including drought, temperature, deterioration due to erosion and other factors, and increasing salinity due to evaporation and/or irrigation practices. At the same time the human population is growing and in many regions high-quality agricultural land is decreasing due to the expansion of urban areas [1].

Salinity is inhibitory to the growth and development of many plants, including most crops [2,3,4,5]. It affects all cellular processes, including disruption of cellular homeostasis, impairment of photosynthesis, mRNA processing, transcription, protein synthesis, disruption of energy metabolisms, amino acid biosynthesis as well as lipid metabolism [6,7,8,9,10]. In response to increasing salt, plant cells activate specific Na^+^ and Cl^−^ ion transporters and adjust the accumulation of cytosolic K^+^ [10,11,12]. Plant cells must also undergo osmotic adjustment, which is accomplished in many ways, including the production of organic osmolytes such as glycine betaine, proline, some sugars, and polyamines, of which most are synthesized in the chloroplast [3,10].

Chloroplasts belong to a family of cellular organelles commonly found in plant and algal cells known as plastids. Green plastids—chloroplasts—are the site where atmospheric CO_2_ fixation occurs through a series of biochemical reactions called the Calvin–Benson cycle by utilizing the energy produced by the light reactions of photosynthesis [13]. Elevated salinity levels affect many cellular processes, including photosynthesis, the major function of chloroplasts. The presence of salt in the soil may cause both osmotic and ionic stresses [14], which may hinder photosynthesis through the diffusional (stomatal, mesophyll and boundary layer resistance to CO_2_) and/or non-diffusional (photochemical and biochemical) limitations of carbon fixation [6,15,16,17,18,19,20]. Salinity exposure is also known to decrease the chlorophyll content in many plants [21,22]. However, salt-resistant plants, particularly those with a C_4_ mechanism, may overcome the inhibitory effect of salinity on CO_2_ fixation more effectively [6,23].

In general, when plants are exposed to salt stress, the very first response is osmotic shock followed by induction of stomatal closure. Stomatal closure, in turn, limits photosynthetic capacity by the restriction of CO_2_ supply. However, research has shown that increasing the external CO_2_ concentration under salt stress did not lead to an increase in photosynthesis rates in many cases. This observation suggests the involvement of some non-stomatal components in photosynthesis reduction under salinity, such as overproduction of reactive oxygen species (ROS) and the depletion of K^+^ inside plant cells due to the accumulation of Na^+^ [24,25]. This results in the disruption of ionic homeostasis in chloroplasts.

Besides CO_2_ fixation, thylakoid reactions are also affected by salinity [6,18,26]. The most commonly studied parameters in this context are the maximum quantum efficiency of the PSII reaction centers (*F_v_/F_m_*), quantum efficiency of PSII (ΦPSII), non-photochemical quenching (NPQ), photochemical quenching (qP) and electron transport rate (ETR), which defines the overall performance of plants under different stresses [27]. Salt-resistant plants are known to possess resilient thylakoid reactions to overcome salinity effects such as photodamage [28] and protection of the reaction centers [29]. This may include protective mechanisms such as cyclic electron flow, photorespiration in C_3_ plants and regulation of NPQ [18,30]. CO_2_ fixation and thylakoid reactions of photosynthesis take place in thylakoids and the stroma of the chloroplast, providing the essential carbon skeleton for growth, energy for driving various metabolic reactions as well as the biosynthesis of different metabolites. Salt-induced toxicity negatively affects all these processes, resulting in poor plant growth and reduction in yield. Chloroplasts are also major reactive oxygen species (ROS) production sites at the reaction centers of PSII and PSI, where charge separation occurs, and the electron transport chain (ETC) from PSII to PSI are highly sensitive to salt-induced toxicity under which ROS production is further increased [31]. Higher concentrations of ROS cause oxidative damage to membranes, lipids, nucleic acids, proteins and some photosynthetic enzymes, resulting in reduced CO_2_ fixation, slower plant growth and consequently low crop yields. The ROS-scavenging system includes both enzymatic and non-enzymatic antioxidants that prevent oxidative damage. Therefore, manipulation of the components of this system holds great implications for improving the photosynthetic rates under salt stress in crop plants. This has been tested by overexpression of Cu/Zn superoxide dismutase (SOD) in the chloroplasts of tobacco [32,33] and Chinese cabbage [34]. Since chloroplasts are largely under the control of nuclear gene expression for growth and metabolic activities, chloroplasts have evolved a sophisticated signaling network to coordinate with the nucleus to control gene expression and maintain the balanced expression of genes in the two compartments. Chloroplasts also act as global sensors relaying changes in their own developmental status as well as in the environmental conditions, including light intensity and stresses to the nucleus. As a result, the nucleus adjusts the expression of its genes to ensure optimal plant performance under changing environmental conditions [35]. Until recently, this chloroplast–nucleus communication has been largely viewed as bilateral, ignoring the pivotal role of chloroplasts in adjusting gene expression and metabolic processes that affect photosynthesis and ultimately crop yields.

In this review, we discuss the effect of salt stress on chloroplasts, their structures, and various biochemical reactions occurring in them. We also compare the differences in how chloroplasts of glycophytes and halophytes respond to salinity stress.

## 2. Effects of Salinity on Chloroplast Ultrastructure

### 2.1. Changes in Chloroplast Structure in Plants

Chloroplasts are roughly 1–2 μm thick and 5–7 μm in diameter. They are enclosed in a chloroplast envelope, which consists of a double membrane with outer and inner layers; the space in between is called the intermembrane space. A third, internal membrane, extensively folded and characterized by the presence of closed disks (or thylakoids), is known as the thylakoid membrane. In higher plants, the thylakoids are arranged in tight stacks called grana. Grana are connected by stromal lamellae extended from one granum through the stroma into a neighboring granum. The thylakoid membrane envelops a central aqueous region known as the thylakoid lumen. The space between the inner membrane and the thylakoid membrane is filled with stroma, a matrix containing dissolved enzymes, starch granules and copies of the chloroplast genome [36].

Several changes have been associated with chloroplast structure in response to environmental factors and the availability of water and minerals [37]. These include modifications in the lamellar organization, resulting in chloroplast shrinkage [37], swelling of chloroplast lamellae and an unrecognizable grana structure under highly saline conditions [38]. Some plants, such as *Atriplex* spp., may undergo lipid deposition to counter the harmful effects of salt-induced toxicity [39,40]. In some instances, starch accumulation under high salinity has also been reported, such as in chloroplasts of wheat cultivars, which was related to damage to the sucrose-phosphate synthase in the cytosol, triggering the triose-phosphate pathway towards starch synthesis [41]. Changes in the ionic composition of starch-degrading enzymes may also be linked with excessive starch deposition [42]. Under saline conditions, reactions involving starch and sucrose biosynthesis are also known to be regulated by changes in the orthophosphate concentration [43,44]. Stress-induced destruction of the chloroplast envelope and an increase in the numbers of plastoglobuli in thylakoid membranes have also been reported in cucumber leaves [45]. Accumulation of starch grains in the chloroplasts of *Thellungiella* and tobacco plants is known to play an important role as osmotica in maintaining the structural integrity of the chloroplasts [46].

### 2.2. Changes in Ultrastructure of Chloroplasts in Glycophytes and Halophytes

Salt stress-induced alterations in the structure of chloroplasts or thylakoid membranes have been extensively examined in various salt-sensitive plants [47,48] as well as in facultative halophytes [49]. Swelling of thylakoids under salt stress (~200 mM NaCl) was reported in rice [50]. However, recent 3D analysis confirmed that rice chloroplasts became spherical under salt stress without any changes in the overall chloroplast volume [51]. Contrasting observations regarding chloroplast volume have been reported among different species. For example, chloroplasts of salt-sensitive cultivars of wheat exhibited an increase in volume possibly due to changes in the ionic composition of the stroma [42]. Spinach chloroplasts showed a decrease in volume with concomitant changes in light-scattering during electron transport [47]. Arabidopsis seedlings grown in the presence of salt also exhibited swollen chloroplasts with less developed granum structures [41]. Changes in the thylakoid ultrastructure of potato [52] and maize [53] under salinity have been previously related to perturbed ion homeostasis in chloroplasts.

In the case of halophytes, salt entry into the chloroplast stroma may be critical for grana formation and photosystem II activity, as halophytes have been reported to accumulate more chloride (Cl^−^) than glycophytes and use sodium (Na^+^) in different functions [54]. Chloroplast swelling of *Atriplex* leaf cells at 345 mM NaCl appeared to be a likely result of the osmotic effect of salinity while few changes were reported in the chloroplasts of hair cells [40]. Similarly, distinct thylakoid swelling in *Thellungiella* under saline conditions (400 mM NaCl) was attributed to the disturbance in osmotic equilibrium [54]. Other notable changes in halophyte chloroplasts include the formation of ‘slim spindle-shaped’ grana with a clear stromal matrix in the halophyte *Kandelia candel* at 400 mM and increases in the plastoglobuli numbers at 600 mM NaCl with intact grana thylakoids [49]. In *Bruguiera parviflora*, no major alterations in the structural integrity or absorption characteristics of the thylakoid membranes were noted at 400 mM NaCl; however, a reduction in antenna size, as well as cytochrome (Cyt) *f* and Cyt *b*_6_ were observed [55].

### 2.3. Changes in the Chloroplast Ultrastructure of C_4_ Plants

Plants with C_4_ photosynthesis possess chloroplasts both in the bundle sheath cell (BSC) and the mesophyll cell (MC). Both of these chloroplast types, BSC and MC, have been reported to respond differently to salt stress. C_4_ plants are further divided into three subtypes, namely, NADP-malic enzyme (NADP-ME), NAD-malic enzyme (NAD-ME) and phosphoenolpyruvate carboxykinase (PCK) types, with peculiar leaf anatomical characteristics [56]. The NADP-ME type 4 species showed little damage to BSC chloroplasts compared to MC chloroplasts [57]. The BSC chloroplasts showed the development of grana when exposed to salt stress [53,58]. On the other hand, thylakoid swelling and disruption of envelopes in MC chloroplasts of both the NAD-ME and PCK types were observed under saline conditions [57]. It has also been reported that exposure to salinity enhanced granal development in BSC chloroplasts and appressed thylakoid density [57]. However, granal development in the NAD-ME and PCK type species is not as pronounced as in the NADP-ME type species. It is believed that granal development in BSC chloroplasts could compensate for the loss of PSII activity in MC chloroplasts under salt stress [53]. In glycophyte plants of the NADP-ME and NAD-ME subtypes, salt stress causes grana unstacking in MC chloroplasts but induces grana formation in BSC chloroplasts. Interestingly, in halophytes of the same subtypes, the grana are constitutively present in BSC chloroplasts and the unstacking of grana is absent in MC chloroplasts [53,58].

### 2.4. Effects of Salinity on Chloroplast Multiplication

Although the number of chloroplasts per leaf cell varies from a few to hundreds [59], they may occupy more than half of the cytoplasm volume in mesophyll cells [60]. Salinity may increase the number of chloroplasts per cell, e.g., in spinach, sugar beet [61], wheat [62], *Arabidopsis* [63] and *Thellungiella halophila* [46]. Bose et al. [29] proposed that halophytes have a greater capacity to increase chloroplast number than glycophytes under salinity, which may help in storing Na^+^ and Cl^−^ without compromising photosynthesis [63]. Increases in succulence help in cell expansion and thus enable housing more chloroplasts [29]. Halophytes can accumulate over 20-fold higher Na^+^ levels in chloroplasts compared to glycophytes [63,64,65,66,67,68]. In addition to compensating for reduced photosynthesis, increases in chloroplast number may also contribute to increased energy demands for osmotic adjustment and ion homeostasis under salinity [29]. Although information about the genes involved in binary fission of chloroplasts is plentiful [68,69,70], the detailed effects of salinity on the mechanism/regulation of chloroplast multiplication, particularly among halophytes and glycophytes, are limited.

## 3. Effects of Salinity on Transport across Chloroplast Membranes

Most of the nuclear-encoded proteins destined for chloroplasts are synthesized as ‘cytosolic preproteins’ and imported by a major pathway consisting of transmembrane protein complexes or channels, ‘*Toc*’ (translocons on outer chloroplast) and ‘*Tic*’ (translocons on inner chloroplast). The ‘*Toc*’ transmembrane channels import larger molecules (including nuclear-encoded proteins) while the ‘*Tic*’ complexes are more restrictive, with transport limited to targeted proteins [71]. Located at the interface between the stroma and the cytosol, the envelope is also the site for the transport and exchange of ions and metabolites required for the integration of the plastid metabolism within the plant cell. In general, chloroplasts harbor three types of membranes and each of them is equipped with a unique set of ion channels and transporters enabling the transport of nutrients, solutes, and metabolites in and out of it. This is achieved by coordinated regulation of a variety of transport systems located in chloroplast membranes, such as porins, solute channels, ion-specific cation/anion channels and various primary and secondary active transport systems [29].

### 3.1. Protein Transport across Chloroplast Membranes

The chloroplast proteome comprises 3000 different proteins, including components of the photosynthetic apparatus, which are highly abundant [72]. Most chloroplast proteins are nuclear-encoded, synthesized in the cytosol, and their import is mediated by multiprotein complexes in the envelope membranes that surround each organelle. The *Toc* complex mediates client protein recognition and early stages of the import. The *Toc* apparatus is regulated by the ubiquitin-proteasome system (UPS) in a process controlled by the envelope-localized ubiquitin E3 ligase SUPPRESSOR OF PPI1 LOCUS1 (*SP1*) [73]. Salinity stress depletes the *Toc* apparatus by enhancing the expression of *SP1*, which in turn may result in the suppression of photosynthesis activity [74].

### 3.2. Ion Transport across Chloroplast Membranes

The proper ionic (K^+^, Na^+^, Cl^−^) balance is essential to control chloroplast volume [73]. For example, Cl^−^ influx from stroma to the lumen is required for thylakoid swelling, while lumen shrinkage is due to K^+^ (or Na^+^) efflux from the lumen to the stroma [75]. The outer membrane is not freely permeable to ions as some porins (OEP23, OEP37) are reported to have high cation selectivity [76], although information regarding their role in plant salt tolerance is lacking. The literature reports several nucleus-encoded candidate ion channels and transporters that regulate Na^+^, K^+^ and Cl^−^ transport through the chloroplast envelope and thylakoid membranes [75,76,77,78,79]. A several-fold increased Na^+^ and Cl^−^ concentration in barley chloroplasts under salt stress has been reported [76]. Slabu et al. [79] reported that salt-induced damage in broad bean chloroplasts is due to the accumulation of Na^+^ and not of Cl^−^ or K^+^. In contrast, salt toxicity and inhibition of photosynthesis in soybean were found associated with the hyperaccumulation of Cl^−^ but not that of Na^+^ in chloroplasts [80,81].

### 3.3. Chloroplast Trafficking of Ions in Glycophytes vs. Halophytes

Halophytes preferentially accumulate ~20-fold higher Na^+^ levels than glycophytes [64,65,67]. This high ion level is known to have some effect on chloroplast functions [63,64,67], especially in the case of CAM and C_4_ plants [81]. The Na^+^ contribution in the transport of pyruvate [82,83], ascorbate [84] and phosphate [85] into chloroplasts has been reported but the effect of salt stress on transport requires further elaboration. Salt stress induces K^+^ loss from chloroplasts in both glycophytes and halophytes. Chloroplasts isolated from halophytes revealed better tolerance to high Na^+^ (100 mmol L^−1^ Na^+^) and low K^+^ (50 mmol L^−1^ K^+^) in the cytosol than chloroplasts of glycophytes [86]. Likewise, halophytes accumulate more Cl^−^ than glycophytes under low salt conditions (≤1 mmol L^−1^ Cl^−^), while at higher salinities some halophytes maintain steady Cl^−^ concentrations, and others show a slight increase within the chloroplasts [63,65]. These findings indicate that halophytes have mechanisms to regulate the Cl^−^ concentrations; however, the candidate transporters for Cl^−^ regulation during salt stress remain uncharacterized.

#### 3.3.1. Aquaporins and Non-Selective ion Channels

Aquaporins (PIP2;1, PIP2.3, PIP2;7, PIP1;3 and PIP1;2) are reported on the chloroplast membrane [77,78]. Expression of both PIP2;1 and PIP2;7 is altered by salinity [87]. Some aquaporins also have the ability to transport ions [88], but little is known about their function/regulation.

Non-selective ion channels include mechanosensitive channels (MSL2 and MSL3) that help reduce chloroplast swelling during hypo-osmotic conditions by releasing ions from the stroma [89]. In general, the ion selectivity of MSLs varies from non-selective to Cl^−^, K^+^, Na^+^ or Ca^2+^ selective channels [75].

#### 3.3.2. Na^+^, K^+^ and Cl^−^ Transporters

Sodium ions (Na^+^) can be transported into chloroplasts through an inner envelope membrane-localized Na^+^-dependent pyruvate transporter (*BASS2*) that is abundantly found in halophyte species compared to glycophytes [82]. Introduction of a halophyte BASS2 gene into glycophyte chloroplasts resulted in improved salt tolerance [83]. The inorganic phosphate transporters (thylakoid membrane-localized PHT4;1 and inner envelope localized PHT4;4 and PHT4;5) can use Na^+^ or H^+^ as a co-transporting ion [79], thereby changing the Na^+^ concentration inside the chloroplasts. The existence of the Na^+^/H^+^ antiporter (NhaD; hereafter NHD)-type transporters at the chloroplast membrane mediating Na^+^ efflux from the stroma was also reported in a halophytic tree, *Populus euphratica* [82]. In Arabidopsis, salt stress did not alter the expression of *NHD1* but silencing *NHD1* resulted in high chloroplast Na^+^ and poor growth and photosynthetic performance [67]. In contrast, analysis of *Mesembryanthemum crystallinum* (a halophyte) under salt stress showed an increase in *NHD1* expression that resulted in higher Na^+^ accumulation, indicating the involvement of *NHD1* in Na^+^ import into the chloroplasts instead of Na^+^ export [41]. Such opposite regulation of ion transport mechanisms requires further investigation for a more complete understanding of the salt tolerance mechanisms.

Two K^+^ efflux antiporters (*KEA1 and KEA2*) located at the membrane of Arabidopsis have been suggested to function as K^+^/H^+^ exchangers mediating K^+^ export out of the stroma [90]. The Arabidopsis double loss-of-function *kea1kea2* mutant showed better growth under salt stress as compared to the wild type, due to low K^+^ efflux in the mutant resulting in increased K^+^ retention as well as maintenance of pH in the stroma leading to improved photosynthetic performance and growth [91]. Arabidopsis *KEA3*, located in the thylakoid membrane, has been suggested to import K^+^ into the lumen in exchange for H^+^ [91,92] and support in PSII quantum efficiency and CO_2_ assimilation under low light [93]; however, no information is available regarding *KEA3* function during salt stress.

**Figure 1:**
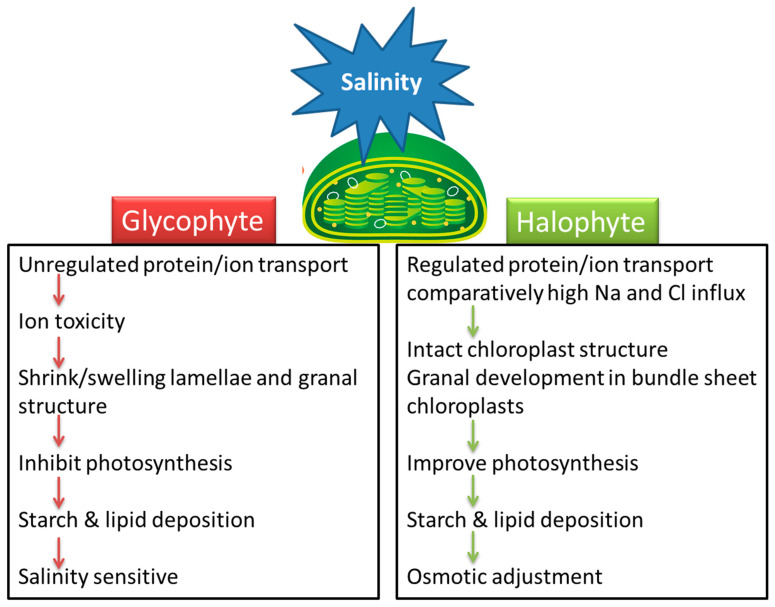
A model that summarizes the effects of salinity stress on chloroplasts in salt-sensitive (glycophyte) and salt-tolerant (halophyte) plants.

Electrophysiological studies have shown the existence of Cl^−^ permeable channels in the chloroplast envelope and thylakoid membranes [75]. A bestrophin-like protein from Arabidopsis has been discovered and shown to alter PMF portioning by functioning as a voltage-dependent Cl^−^ channel (*AtVCCN1*) on the thylakoid membrane [93]. The effects of salinity on chloroplasts are summarized in the model in Figure 1.

## 4. Effect of Salinity on Osmotic Adjustment in Chloroplasts

### 4.1. What Is Osmotic Adjustment and How Is It Achieved?

Hyper-osmotic stress due to salinity is well-known in plants and bacteria and may cause disrupted cell metabolism, turgor loss and growth arrest. However, an adaptive mechanism for water retention exists among organisms under stressed environments whereby they increase their osmolality, a phenomenon commonly termed as ‘osmotic adjustment’ [94]. Increases in osmolality are achieved by either of the following three mechanisms: (1) micro-organisms, such as bacteria or yeast, accumulate a range of osmolytes or compatible solutes available from the external medium; (2) plants activate genes for de novo synthesis of organic osmolytes (so-called ‘compatible solutes’), such as glycine betaine, proline, sugars, polyols, etc.; and (3) plants regulate ion flux across cellular membranes [20,95].

### 4.2. Localization, Trafficking and Functions of Organic Osmolytes in Membrane-Bound Organelles

Among the organic osmolytes, glycine betaine (GB), sugars (mannitol, sorbitol and trehalose), polyamines and proline are the most important and are accumulated under abiotic stresses and confer tolerance to cells without interfering with the cellular machinery of the plant [96]. Of these osmolytes metabolism of proline (PRO) depends upon two important enzymes, catalyzing its synthesis from glutamate in the cytoplasm or chloroplast and two enzymes catalyzing proline catabolism back to glutamate in the mitochondria along with an alternative pathway of its synthesis via ornithine [97]. During water deficit or physiological drought under salinity PRO is known to protect the photosynthetic apparatus as well as in cytokinin-dependent photorespiration [98]. Studies on other osmolytes suggest that sugar alcohols, such as sorbitol and mannitol, and quaternary ammonium compounds, such as GB and their precursors, are highly localized in chloroplasts [99,100] and are somehow involved in protecting the photosystem (PSII) and membrane proteins against ROS under stress conditions [95,101,102]. The impairment of thylakoid membranes that results from salt stress may be alleviated by GB probably via protection and stabilization of the protein complexes as well as changes in lipid composition of the thylakoid membrane, thereby improving photosynthesis [102]. The accumulation of GB in higher concentrations in the chloroplasts of young leaves suggests that these are the main sites of its biosynthesis [98,103] from where it is translocated to other plant parts via phloem [104]. Sugar alcohols and polyols, such as mannitol, sorbitol, etc., regulate osmotic balance by sequestering Na^+^ in the vacuole or apoplast, thus protecting membranes against drought [105] and salt stress [106]. These osmolytes also scavenge ROS, particularly hydroxyl radicals that do not require high concentrations of osmolytes as needed for osmotic adjustment [97]. This leads to the conclusion that such compounds may be more important in ‘osmoprotection’ rather than ‘osmotic adjustment’.

### 4.3. Are Osmolytes Compatible for Osmotic Adjustment in Planta?

The classical concept of osmotic adjustment via accumulation of organic solutes in non-halophilic organisms still prevails [107,108] though it has been challenged by many physiologists [97,109,110]. A major shift in energy balance usually causes severe losses in growth yields of crop plants at the expense of other metabolic processes, raising the serious question of whether osmolytes are compatible in a real sense. Physiologists argue that conventional water retention under saline stress is not directly related to the contribution of organic solutes for many reasons. The first reason is the concentration of organic osmolytes, which seems to be too low compared to the inorganic solutes in cells. For instance, 3–10 mM in plants contributes less than 3% [111,112], while ~120–150 mM glycine betaine (GB) in plants contributes <50%, often ranging between 10 and 30% of the total cell solutes [112]. Even if it is assumed that most of the osmolytes are contained in the cytosol and chloroplasts (collectively constituting 10–15% of the cell volume) compared to a larger vacuolar fraction (~85%), this seems low given that 500–600 mM concentrations of Na^+^ alone exist within the vacuole [99]. Osmolyte concentrations (GB in particular) between 200 and 300 mM may be sufficient to prevent cytoplasmic dehydration, thereby achieving osmotic adjustment. In some of the succulent halophytes (which accumulates up to 1000 mM Na^+^ and Cl^−^), ~200 mM plant water GB was reported in *Suaeda fruticosa* and about 600 mM in *Haloxylon stocksii* (sensu lato *recurvum*) under extreme saline conditions, which are exceptional as in other plants, including *Halopyrum mucronatum* and *Atriplex stocksii* (sensu lato *griffithii*), GB ranged between 100 and 150 mM [113]. The second reason for not considering organic osmolytes as ‘compatible’ is the cost of their synthesis, which is too high. For instance, 30–109 molecules of ATP may be required for osmolyte synthesis compared to one molecule of ATP for one K^+^ and two Cl^−^ in bacteria [114], while plants require approximately 41 molecules of ATP for proline, 50 for glycine betaine and 52 for sucrose [115]. Thirdly, the synthesis of such organic solutes is very slow, often ranging from hours to many days while plants growing in water-stressed environments require rapid turgor recovery [102]. Moreover, salt-sensitive genotypes of many crop plants, e.g., rice, wheat, barley, etc., accumulate comparatively higher amounts of osmolytes than salt-tolerant varieties, which also creates ambiguity in the role of osmolytes in achieving osmotic adjustment [81,109,116].

### 4.4. Effects of Osmolytes on Organelles

Although the osmotic adjustment is based on the notion that osmolytes should not interfere with other metabolic processes, some studies on exogenous application of osmolytes suggest their toxic effects on plant growth as well as cell organelles [116,117]. Application of some L-amino acids (L-proline, L-alanine, etc.) in millimolar concentrations caused growth inhibition in suspension cultures of *Nicotiana silvestris* [116]. In another instance, a disruptive effect of PRO on the ultrastructure of chloroplasts in *Arabidopsis thaliana* was linked to feedback inhibition of its synthesis due to over-reduction of the photosynthetic electron acceptor pools [117]. In the same plant, exogenously supplied PRO was thought to have increased the rates of mitochondrial electron transport, resulting in elevated levels of ROS causing subcellular damage [117]. On the contrary, endogenous PRO did not seem to have a negative impact on the ultrastructure of chloroplasts and mitochondria in transgenic tobacco, indicating that this level of PRO had no toxic effects [118]. Though the assumption of osmolyte toxicity is largely inconclusive, it seems that plants treated with exogenous application of osmotica may suffer from an ‘overdose’ compared to their endogenous levels. In fact, in certain cases, exogenous application (both foliar as well as through the rooting medium) of osmolytes such as GB, PRO, inositol, and mannitol have indicated stress alleviation in many plants [29,119]. Exogenous application of osmotica, such as GB, may also enhance the membrane integrity of chloroplasts and also increase PS II efficiency [97,98], suggesting an osmoprotective role. Experiments on exogenous application of osmolytes have intrigued geneticists to manipulate the biosynthetic pathway of compatible solutes to enhance salt tolerance as osmolyte accumulation is often controlled by only one gene [102].

### 4.5. Possible Role of Osmolytes in Ion Regulation

Although the published literature has contradicting reports on the role of osmotic adjustment via osmolytes for maintaining turgor, recent patch-clamp studies suggest that osmolytes may have a significant contribution in regulating ion transporters such as K^+^ outward rectifying channels (KORs), though this requires further investigation [120]. Thus, ion regulation via osmolytes may prove to be an important aspect in conferring salt tolerance. In plants, K^+^ appears to be the most abundant cation in the cytosol (100–150 mM), which may account for osmotic adjustment [121], though under stressed conditions, the electrochemical gradients may lead to the loss of K^+^. In halophytes, Na^+^ and Cl^−^ seem to play a major role in osmotic adjustment [105]. Of these, Na^+^ may enter the cell passively and could be used as a cheap osmoticum for maintaining cell turgor. Since Na^+^ is toxic and may cause an imbalance in the cytosolic K^+^/Na^+^ ratio and interferes with cell metabolism, its efficient sequestration in the vacuole is thus essential. Pumping of one mole of Na^+^ against the electrochemical gradient requires only 3.5 mol of ATP compared with 30–50 mol of ATP for one mole of organic osmolyte [115]. As mentioned above, some of the sugar alcohols and polyols regulate osmotic balance by sequestering Na^+^ in the vacuole or apoplast. It appears that osmotic adjustment is collectively achieved by maintaining a balance between ion regulation, synthesis and accumulation of organic solutes, as well as maintenance of K^+^ in the cytosol [122].

## 5. Effects of Salinity on Function and Protection of Photosystems

Under saline conditions, decreases in CO_2_ assimilation via the Calvin cycle accompany a decrease in photochemical electron sink, which in the presence of light impacts the functioning/efficiency of photosystems [31]. In some sensitive plants such as olives, decreases in the *F_v_/F_m_* ratios indicate the incidence of photodamage under saline conditions [35]. Likewise, increases in salinity resulted in a gradual decrease in activities of PSI and PSII in four rice cultivars [123]. However, unchanged *F_v_/F_m_* hints towards sustained PSII under saline conditions [124], such as in the Mangalamahsuri variety of rice [125]. PSII-mediated electron transport increased in low salinity followed by a decrease at high salinity in the halophyte *Bruguiera parviflora* [55]. In other instances, inhibition of de novo protein synthesis, especially of the D1 protein, indicated a lack of efficient PS II repair under saline conditions [2,126,127]. A compensation mechanism of PsbO protein induction has been observed in some studies to stabilize the PSII structure under salinity [128]. Among C_3_ plants, salinity reportedly resulted in poor PSII function in glycophytes such as rice and Arabidopsis [41,129] but not in the halophyte *Arthrocnemum macrostachyum* [130,131]. Several tolerant species, including halophytes such as *Sarcocornia fruticosa* [132] and *Atriplex centralasiatica* [133], also employ the xanthophyll cycle for non-photochemical quenching that dissipates excess excitation energy of PSII in the form of heat as a ‘first line of defense’ [31,133], thus preventing the formation of potentially cytotoxic reactive ROS. The xanthophyll cycle enzyme violaxanthin de-epoxidase consumes NADPH, which if accumulated may cause the over-reduction of reaction centers, and thereby enhance ROS (especially superoxide) formation [134]. Hence, the timely induction of the xanthophyll cycle may protect plants under stressful conditions in multiple ways. Many halophytes are reported to exhibit reversible midday photoinhibition of PSII activity to limit excitation of the PSII reaction centers [130,134]. This mechanism also minimizes the possibility of ROS formation in salt-stressed plants under high light and is considered an important ecophysiological adaptation to salinity [31]. A decrease in the antennae size due to decreased chlorophyll content was also observed in *Arthrocnemum macrostachyum* to limit PSII excitation [132].

PSI is reportedly more stress-resistant than PSII and seems to impart salt tolerance by increasing cyclic electron flow to generate ATP while avoiding the accumulation of toxic-reducing species [135,136,137]. Information about PSI in halophytes is scarce. PSI reaction center subunit IV protein (PsaE) was upregulated under salinity in wild halophytic rice *Porteresia coarctata* but not in conventional sensitive rice [137]. Similarly, salinity treatment caused an increase in PSI transcripts in *M. crystallinum* [138]. Formation of ATP via cyclic electron flow around PSI helped to prevent overaccumulation of Na^+^ in chloroplasts of soybean [139].

Cultured plant cell lines have also been utilized to study salt-adapted tobacco cells [140,141]. Heterotrophic tobacco cells adapted to grow at 428 mM NaCl showed elevated levels of chlorophyll and lower levels of starch along with increased CO_2_ fixation, oxygen evolution and photorespiration, compared to unadapted cells [140]. This was coupled with higher levels of PS-I- and PS-II-associated proteins, including Rubisco. These cells were found to have acquired a significant level of salt-tolerant photosynthetic competence [140]. Further analysis showed that oxygen evolution and CO_2_ fixation were more resistant to inhibition by NaCl in the salt-adapted cells [141].

## 6. Effects of Salinity on CO_2_ Assimilation Enzymes

Information on the effects of salinity on chloroplast CO_2_ assimilation enzymes is limited among halophytes. Generally, CO_2_ assimilation reactions are considered more sensitive to salinity than photochemical reactions of photosynthesis [31]. Several studies have reported that salinity generally inhibits many enzymes of the Calvin cycle [137,142,143].

Ribulose-1,5-bisphosphate carboxylase/oxygenase (Rubisco) is the key photosynthetic enzyme that catalyzes the fixation of atmospheric CO_2_ in plants during the Calvin cycle [144]. It is the most abundant protein in leaves that accounts for 30% (C_4_ plants) to 50% (C_3_ plants) of total soluble protein in leaves [145]. In C_3_ plants, it is localized in all chloroplasts while in C_4_ plants with Kranz anatomy, Rubisco is localized specifically in the bundle sheath but not mesophyll chloroplasts [65]. In single-cell C_4_ species, Rubisco mRNA could be targeted to the proximal or central compartment of chloroplasts [146]. The activity of Rubisco was mostly examined by direct measurement of the enzyme activity or protein levels and measurement of its carboxylase activity (V_cmax_) [12]. Salinity exposure causes a decrease in Rubisco activity in most plant species regardless of C_3_ or C_4_ type [19,146,147,148]. In addition, the Rubisco levels also decreased under saline conditions in both halophytes and glycophytes. For example, salinity caused an inhibition (~50%) of Rubisco activity in maize, a glycophyte, and in *Atriplex spongiosa*, a halophyte [149]. In some other instances, Rubisco activity was improved both in either low [13,20] or high salinity [150]. Rubisco activity also depends on the function of a supporting enzyme, Rubisco activase, which revitalizes the active sites of Rubisco by removing inhibitory sugar phosphates [151,152]. The enhanced activity of Rubisco activase was found in rice as well as in many halophytes, such as *S. salsa* [143] and *Thellungiella salsuginea*, under saline conditions [153]. More efficient Rubisco activation was found in *T. salsuginea* compared to *Arabidopsis thaliana* [153].

Chloroplastic fructose-1,6-bisphosphatase is considered a metabolic control point of the Calvin cycle [44,154]. In vitro salt sensitivity of this enzyme was higher in salt-sensitive rice (*Oryza sativa* cv. IR26) than its wild halophytic relative *Porteresia coarctata* [142]. However, the inhibitory effects of salinity could be reversed by preincubation of the enzyme with osmolytes (effectiveness order: polyol>sugars) [142], suggesting a lower level of in vivo inhibition of chloroplastic fructose 1,6-bisphosphatase under salinity in halophytes with higher amounts of osmolytes compared to glycophytes.

Phosphoenolpyruvate carboxylase (PEPC) is the key enzyme of C_4_ photosynthetic metabolism that catalyzes the β-carboxylation of phosphoenolpyruvate to form four-carbon acid oxaloacetate in the mesophyll cells [144,155]. It is considered more sensitive to salinity than Rubisco [149]. Furthermore, PEPC isolated from the halophyte *Atriplex spongiosa* was found more salt-sensitive in the in vitro studies than the one from the glycophyte maize [149]. Contrary to these observations, an increase in PEPC activity was reported in the halophyte *Mesembryanthemum crystallinum* [156] and in the C_4_ species *Bienertia sinuspersici* under salinity [157]. Increased PEPC activity helps concentrate CO_2_ around Rubisco and substantially reduces the incidence of photorespiration, a major cause for growth reduction and ROS formation under environmental stresses in plants.

Pyruvate orthophosphate dikinase is the rate-limiting enzyme of the C_4_ cycle that catalyzes a reversible reaction to regenerate the primary CO_2_ acceptor phosphoenolpyruvate (PEP) [158]. However, its role in C_3_ plants is not fully understood [159]. Pyruvate orthophosphate dikinase is found in both chloroplasts and the cytoplasm irrespective of C_3_ or C_4_ types [160]. In C_4_ plants, it can comprise up to 10% of the total protein pool [161]. Interestingly, both isoforms are encoded by a single nuclear gene [162]. The labeling of the pyruvate orthophosphate dikinase protein was observed both in mesophyll and bundle sheath chloroplasts of kranz type C_4_ plant maize, albeit with higher levels in the latter rather than the earlier-mentioned chloroplasts [163]. In single-cell C_4_ species, pyruvate orthophosphate dikinase mRNA could be targeted to the peripheral or distal compartment chloroplasts [146]. Information about the impacts of salinity on the abundance and activity of this enzyme is scant. Salinity caused an increase in pyruvate orthophosphate dikinase levels in both types of chloroplasts in maize [163]. These enzymes are widely studied and are important for the biochemical reactions of photosynthesis [17,164,165]. Induction of PEP activity would also help maintain C_4_ functionality under salinity stress and facilitate CO_2_ assimilation for biomass buildup and reduce photorespiration, as mentioned above.

### Effects on Salinity on the Gas Exchange Ecophysiology of Photosynthesis

The effects of salinity on photosynthetic synthetic gas exchange, which eventually supports CO_2_ assimilation at the chloroplast level, varies not only among species but also depends on the magnitude of the salinity. For instance, the net CO_2_ assimilation rate (*P_N_* or *A*) and stomatal conductance (*Gs*) in sugar beet improved under low (75 mM NaCl) salinity while high (250 mM NaCl) was inhibitory [166]. An increase in *P_N_* but not in transpiration (*E*) under low salinity resulted in improved water-use efficiency (WUE) in sugar beet plants [166]. Salinity stress decreased the *P_N_* and *Gs* in wild-type wheat plants [24]. *P_N_* and *Gs* increased transiently at 200 mM NaCl in comparison to controls and 400 mM NaCl in the halophyte *Sesuvium portulacastrum* [167]. Similarly, in many other halophyte species, such as *Arthrocnemum macrostachyum* (in up to 510 mM NaCl) [133] and *Atriplex portulacoides* (200 mM NaCl) [168], low to moderate salinity improved *P_N_*. In contrast, salinity exposure resulted in decreased *P_N_* and *Gs* in the halophytes *Panicum antidotale* [20] and *Aster tripolium* [169]. Hence, impacts of salinity not only vary among glycophytes but also halophyte species. In many cases, decreased *Gs* improves the WUE of plants under stress conditions as a trade-off at the expense of *P_N_*. For instance, in *Sarcocornia fruticosa*, increased WUE accompanied a decline in *P_N_* [170]. Similarly, many halophytes exhibit C_4_ and CAM modes of photosynthetic CO_2_ assimilation, which not only reduce wastage of photosynthetic energy through photorespiration but also decrease the consequent H_2_O_2_ (a common ROS) production at the peroxisome level [31,171].

## 7. Effects of Salinity on Chloroplast ROS Homeostasis

Exposure of plants to salinity results in a reduction in CO_2_ assimilation rates, which in turn leads to the overreduction of PSII along with diversion of electrons to molecular oxygen, which generates reactive oxygen species (ROS), particularly singlet oxygen [31,172,173]. In photosynthesizing leaves, chloroplasts are the major site for ROS production during the daytime [174]. In C_3_ plants, photorespiration resulting from the oxygenase activity of Rubisco in chloroplasts is another source of ROS generation in peroxisomes [31]. Salinity-induced stimulation of electron flow to molecular oxygen has been reported in several plant species [31,175,176]. Major ROS produced in chloroplasts include singlet oxygen (^1^O_2_), superoxide radical (O_2_^•−^), hydrogen peroxide (H_2_O_2_) and hydroxyl radical (^•^OH) [31,173]. Since detection of radicle-type ROS is difficult, most studies examine H_2_O_2_ (non-radicle ROS) formation following salinity exposure [31]. In addition, studies on ROS formation in isolated chloroplasts, particularly of halophytes, are very limited. Wiciarz et al. [153] reported that isolated thylakoids from a halophyte *Thellungiella salsuginea* produced higher H_2_O_2_ levels than the model glycophyte *Arabidopsis thaliana*. However, when both plant types were exposed to salt stress, even at the low level of 100 mM NaCl, Arabidopsis produced a higher H_2_O_2_ than *T. salsuginea* and at a 300 mM NaCl concentration. Similarly, a substantially higher H_2_O_2_ level was observed in chloroplasts of wild salt-tolerant tomato *Lycopersicon pennellii* compared to chloroplasts of sensitive tomato *L. esculentum* under stress-free growth conditions. However, under NaCl stress, a decrease in H_2_O_2_ level was noted for wild tomato while the levels were increased in the sensitive species [171]. This indicates that halophyte species have efficient mechanisms to control the production of ROS or detoxify them compared to glycophytes, either through the dissipation of excess excitation energy to alternative electron sinks, such as the plastid terminal oxidase [29,172,173,174,175,176,177,178,179,180] (PTOX) or ROS-scavenging system [31,174]. Alternative electron sinks not only provide ‘safety valves’ for the efficient functioning of the photosynthetic machinery but also act as an ‘avoidance’ tool for control of ROS formation. Tightly regulated levels of ROS are now acknowledged as ‘signals’ for the regulation of different plant processes, including the defense/tolerance response of plants [3,99]. For instance, ROS modulate the function of some plasma membrane ion transporters, such as those regulating cytosolic Na^+^ and K^+^ [177,181,182,183,184,185]. Similarly, a ROS ‘surge’ in response to salinity exposure may also activate chloroplast retrograde signaling pathways [180].

**Figure 2:**
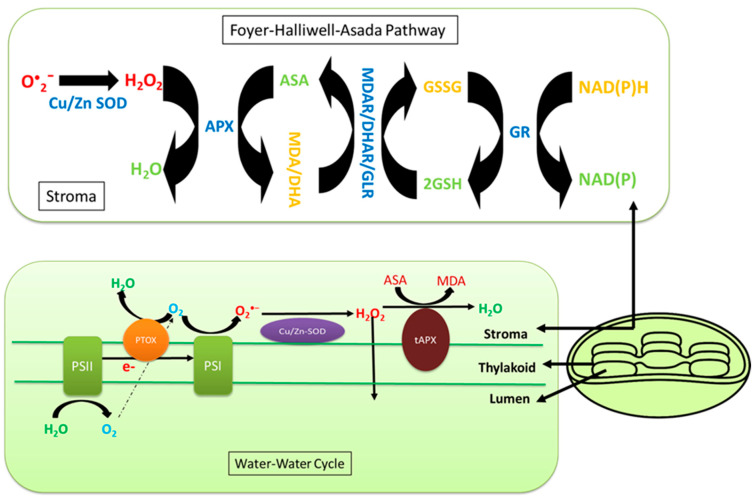
The Foyer–Halliwell–Asada pathway (also known as the ascorbate–glutathione cycle) and the water–water cycle are responsible to quench the superoxide radicles and hydrogen peroxide in the chloroplasts.

In order to prevent oxidative damage due to ROS accumulation, chloroplasts possess many enzymatic and nonenzymatic antioxidants [29,138,182,185]. Key enzymatic antioxidants are superoxide dismutases (SOD), enzymes of the Foyer–Halliwell–Asada pathway (also known as the ascorbate–glutathione cycle), and glutathione peroxidase (GPX), whereas ascorbate and glutathione are common nonenzymatic antioxidants of chloroplasts (Figure 2) [138,182,186]. Antioxidants in various combinations play an important role to keep the levels of ROS in ‘functionally useful’ ranges for signaling various plant processes and stress responses [31]. The water–water cycle is among the key processes responsible for ROS homeostasis in chloroplasts and is essential for salinity tolerance (Figure 2) [31,187]. Ground state molecular oxygen (O_2_) produced during photolysis of water in chloroplasts can accept electrons from excited photosystems, particularly the thylakoid membrane-bound primary electron acceptor of PSI to form O_2_^•−^ through a reaction called the Mehler reaction [188]. The acceptor side of the electron transport chain in PSII may also contribute to electron leakage to O_2_ to generate O_2_^•−^. Thylakoid membrane-bound copper/zinc superoxide dismutase (Cu/Zn SOD) converts O_2_^•−^ into H_2_O_2_, which is finally reduced into the water by the action of thylakoid membrane-bound ascorbate peroxidase (tAPX), thus completing the ‘water–water cycle’ [189]. The Foyer–Halliwell–Asada pathway (also known as the ascorbate–glutathione cycle) in chloroplasts is an extension of the water–water cycle and involves quenching of ROS in chloroplasts by consuming NADPH, which also contributes to relaxing the ‘overreduction of photosystems’ by providing NADP (the final electron acceptor of PSI), and thereby minimizing the chances of further ROS generation (Figure 2) [167,190]. In this pathway, the H_2_O_2_ generated from dismutation of O_2_^•−^ by SOD is neutralized into water by the action of stromal ascorbate peroxidase (APX) using ascorbate (AsA) as the electron donor. Oxidized ascorbate is recycled by monodehydroascorbate reductase (MDHAR) and/or dehydroascorbate reductase (DHAR). The latter consumes glutathione (GSH), which is finally recycled by the action of glutathione reductase (GR) that uses NADPH as an electron donor [167,190,191]. Often, an upregulation of enzymes involved in antioxidant processes is reported in chloroplasts under environmental stresses, with a higher magnitude of tolerance compared to sensitive species [31,181]. For instance, salinity exposure resulted in enhanced activities of SOD, APX and MDHAR in chloroplasts of halophytic wild tomato *Lycopersicon pennellii* compared to conventional sensitive tomato *L. esculentum* [172]. In addition, thioredoxin/peroxiredoxin (Trx/Prx) and glutathione peroxidase (GPX) also reportedly quenched salinity-induced excess H_2_O_2_ in chloroplasts [187]. Lipophilic tocopherol can protect chloroplast thylakoid membranes from oxidative damage [189]. The ^1^O_2_ produced by PSII is mainly detoxified by carotenoids and tocopherols found in the chloroplast membranes [192]. Carotenoids detoxify ^1^O_2_ not only through the xanthophyll cycle (NPQ) but also by direct quenching of ^1^O_2_ [193]. However, some C_4_ plants, especially those with NADP-malic enzyme (NADP-ME) subtypes, lack PSII in their bundle sheath chloroplasts and hence supposedly lack ^1^O_2_ production [194].

## 8. Summary

Soil salinity is one of the major challenges to the sustainable development of agriculture in different parts of the world. Salinity has detrimental effects on plant growth by imposing several constraints. For instance, salt-induced toxicity impairs the normal functioning of the organelles, such as chloroplasts—the green plastids—which house several important biochemical reactions, including photosynthesis. Chloroplast dysfunction as a result of various environmental stresses, including salinity, has been reported to have detrimental effects on plants [173]. Chloroplasts, in addition to being a site of various metabolic reactions, also act as global sensors to sense and communicate the developmental, operational and environmental changes to the nucleus.

Understanding the effect of salinity on chloroplast function and the response of various metabolic reactions to salt stress is necessary for the development of salt-tolerant crops. Little attention has been paid to how salinity affects chloroplasts and the stromal metabolic reactions. Salinity-related changes in the size, number, lamellar organization, lipid and starch accumulation, and trafficking across the chloroplast membrane are dependent on the plant species and its level of salt tolerance. Chloroplast swelling or alteration in thylakoid membranes of glycophytes may be linked with the ionic component of salinity while some halophytes are affected by the osmotic effect of high salinity (Figure 1). Most halophytes either maintain chloroplast structure or enhance grana development under salinity stress. Swelling of thylakoids and disruption of chloroplast envelopes in mesophyll cells along with intact chloroplasts in bundle sheath cells is a general C_4_ response under salinity, irrespective of the subtype.

Halophytes and glycophytes have evolved different pathways to respond to salinity stress. For example, halophytes are much better adapted at maintaining a lower salt concentration in the cytoplasm compared to glycophytes. Likewise, chloroplasts in halophytes seem to have a better antioxidant system than those of glycophytes, and consequently more protected photosynthetic apparatuses under salt stress. Similarly, salinity-triggered starch deposition appears to be a damage symptom in glycophytes but a survival strategy in halophytes. The salinity-induced influx of Na^+^ and Cl^−^ appears beneficial for halophytes but lethal for glycophytes (Figure 1). Accumulation of Na^+^ or Cl^−^ disrupts ionic homeostasis, impairs protein synthesis and interferes with the enzymatic activities of the organelle. However, recent work suggests that the negative effects of these ions on plant health are not because of toxicity per se but are the result of interference with the absorption or metabolism of other essential ions [28]. This view stems from the evidence that K^+^ influx in chloroplasts is reduced with excessive Na^+^ or Cl^−^ accumulation. K^+^ is an essential element for the plant cell and is not only required for chloroplast development but also for pH regulation, maintenance of the electron transport chain and thylakoid restacking [28,29]. Osmolyte synthesis suggests that organic solutes may help in fine adjustment along with ion transport (vacuolar compartmentation) and accumulation of cytosolic K^+^ in stressed environments rather than osmotic adjustment. However, osmolytes are certainly involved in the osmoprotection of membrane transport proteins and the scavenging of ROS. Despite ion regulation and osmotic adjustment, salinity induces many changes in chloroplast functions and signaling.

Chloroplastic CO_2_ fixation is generally more sensitive to salinity than the thylakoid reactions. However, CO_2_ fixation in many halophytes is reportedly less prone to salinity compared to glycophytes. One major evolutionary adaptation that seems to operate in halophytes is the switching of CO_2_ concentration around Rubisco under stressful environmental conditions, including salinity. The reduced photosynthetic efficiency is considered a major salt-induced constraint inhibiting plant growth, and ultimately crop productivity. However, it is not yet clear whether the decrease in photosynthesis is the cause of growth reduction or the reduction in the growth rate causes a decrease in photosynthesis under salt stress. Nevertheless, a reduced rate of photosynthesis leads to higher production of ROS and also triggers the activity of ROS-scavenging enzymes. The higher activity of the ROS-detoxifying enzymes maintains a level of these species in a functionally useful range required for cell signaling. These enzymatic systems are naturally present in plants. Although differences in the activity of these enzymes have been reported in different genotypes, it is believed to be associated with responses such as stomatal closures, reduction in the CO_2_ fixation rates and an increase in photorespiration under stressful conditions [188,194]. Tight regulation of ROS alongside many chloroplastic metabolites also function as ‘putative’ signals for communication between chloroplasts and the nucleus (as well as other organelles) via so-called ‘retrograde signaling’. Despite information on crop and model plants, our knowledge about such signaling in halophytes is still far from full comprehension. Chloroplast functions, including photosynthesis, are integrated with other basic plant metabolic mechanisms of the plant in response to stresses, including salinity, and multiple factors work together to confer tolerance against salinity [195]. These factors include ion regulation that controls uptake and transport of salt and other ions to compartments within the plant cell, synthesis of compatible solutes, antioxidative enzymes and plant hormones and changes in photosynthesis and membranes in the cell [195]. Some of these occur within the chloroplast but are not limited to that location. These mechanisms are quite complicated, and many questions remain unanswered [195,196]. Some of these questions include how the plant senses salinity to initiate the signaling process, the precise details of how salinity stress leads to stomatal closure and growth reduction and the specific targets of ion toxicity in plant cells [196]. While advances are being made, a detailed understanding of the mechanisms behind salt tolerance is not yet clear. A comprehensive understanding of these mechanisms by employing multidisciplinary approaches is necessary for their effective incorporation into salt-sensitive crops for better crop yields under stressful environments. 

## Data Availability

As a review paper all data is available in the work referenced.
